# *Blastocystis* presence alters gut archaeal communities and metabolic functions in Tibetan antelopes (*Pantholops hodgsonii*)

**DOI:** 10.3389/fvets.2025.1744013

**Published:** 2025-12-23

**Authors:** Jin-Wen Su, Si-Yuan Qin, Jian Liu, Cong-Cong Lei, Xiao-Tian Zhang, Wen-Hui Shi, Lin-Hong Xie, Yan Liu, Hong-Bo Ni, Ming-Yuan Yu, Hong-Rui Liang, Ya Qin, Jing Jiang, He-Ting Sun, He Ma, Zhong-Yuan Li, Xiao-Xuan Zhang

**Affiliations:** 1 Guangxi Key Laboratory of Brain and Cognitive Neuroscience, College of Basic Medicine, Guilin Medical University, Guilin, Guangxi, China; 2 College of Veterinary Medicine, Qingdao Agricultural University, Qingdao, Shandong, China; 3 Center of Prevention and Control Biological Disaster, State Forestry and Grassland Administration, Shenyang, Liaoning, China; 4 College of Veterinary Medicine, Jilin Agricultural University, Changchun, Jilin, China; 5 College of Life Sciences, Changchun Sci-Tech University, Shuangyang, Jilin, China

**Keywords:** blastocystis, gut archaea, metagenomics, Tibetan antelope, virus

## Abstract

**Background:**

Archaea are vital members of the gut microbiota, yet their diversity and functions in high-altitude wildlife remain poorly understood. Understanding their ecological roles can provide insights into host health and microbial community dynamics.

**Methods:**

We applied metagenome-assembled genome (MAG)-based approaches to investigate gut archaea in Tibetan antelopes (*Pantholops hodgsonii*) and assess their shifts in the presence of Blastocystis. A total of 463 non-redundant archaeal MAGs were reconstructed and analyzed for taxonomic diversity and functional potential.

**Results:**

The MAGs encompassed 16,189 protein clusters, with over 70% representing potentially novel species, highlighting substantial unexplored archaeal diversity. Alpha diversity showed no significant differences between healthy and *Blastocystis*-present groups, but beta diversity analysis revealed marked community restructuring, including decreased *Methanobacteriota* and increased *Halobacteriota* and *Thermoplasmatota* in the *Blastocystis*-present group. Functional annotation indicated changes in energy and nucleotide metabolism and alterations in carbohydrate-active enzyme composition. Additionally, putative viral sequences were detected within archaeal MAGs, suggesting potential virus-microbe interactions.

**Conclusion:**

Our findings provide novel insights into the diversity and ecological functions of gut archaea in Tibetan antelopes, offering a foundation for future research on their contributions to host health and microbial ecology.

## 1 Introduction

Gut archaea represent a crucial component of the host microbiome, playing essential roles in maintaining intestinal ecological balance, participating in methane metabolism, and contributing to physiological processes closely associated with host health (1, 2). Although extensive studies have investigated gut archaea in humans and various animals, research on archaea in high-altitude species remains limited (3, 4). Prior studies have revealed that archaea can act as hosts for diverse archaeal viruses that shape archaeal population dynamics and genome evolution (5, 6), yet archaeal viruses in animal-associated gut ecosystems remain poorly characterized, particularly in wild or high-altitude mammals. The Tibetan antelope (*Pantholops hodgsonii*), a flagship species endemic to the Qinghai-Tibet Plateau, inhabits an environment characterized by extreme cold and low oxygen levels. Its unique physiological adaptations are thought to shape a distinct gut microbial profile (7–9). However, the specific composition, functional potential, and microbial interactions of archaeal communities in Tibetan antelopes remain largely unexplored.

*Blastocystis* is a common intestinal protist found in the gastrointestinal tracts of both humans and various animals (10–12). *Blastocystis* presence has been linked to gastrointestinal symptoms such as diarrhea, abdominal pain, and irritable bowel syndrome, although evidence remains inconclusive as to whether it acts as a pathogen or a commensal organism. It has also been associated with notable alterations in the gut microbial community structure (10, 11, 13, 14). While the interactions between *Blastocystis* and the gut microbiome have been studied in livestock, primates, and humans, their impact on the gut microbiota of wild animals particularly on archaeal communities remains poorly understood. Protist-archaea interactions have been proposed in gut ecosystems, particularly through metabolic cross-feeding, where protists produce hydrogen that can be consumed by methanogenic archaea (15–17). Such metabolic linkage can shape archaeal growth, community composition, and methane production, making archaea sensitive indicators of protozoan-associated ecological shifts. Given the unique physiological and ecological adaptations of Tibetan antelopes to the high-altitude environment, their response to *Blastocystis* presence may differ significantly from that of other species (18). Thus, exploring the effects of *Blastocystis* presence on the gut archaeal communities of Tibetan antelopes is of great ecological and conservation importance.

In this study, we employed a genome-resolved metagenomic approach to reconstruct a non-redundant archaeal genome catalog from the gut microbiota of Tibetan antelopes. We systematically analyzed the diversity, taxonomic composition, and functional alterations of gut archaea in response to *Blastocystis* presence. Comparative analyses with human and chicken gut archaeal communities further revealed the uniqueness and potential novelty of the archaeal lineages in Tibetan antelopes. Collectively, our findings advance the understanding of archaeal diversity in high-altitude wild animals and provide a new perspective on microbe–protist interactions, with important implications for ecology and evolution.

## 2 Materials and methods

### 2.1 Collection of metagenomic and MAG data from the gut microbiome of Tibetan antelope

In this study, a total of 33,925 MAGs from the gut microbiome of Tibetan antelope (*Pantholops hodgsonii*) were collected, including 26,607 MAGs from the CNGBdb database (Project ID: CNP0001390) and 7,318 MAGs from the Figshare repository (http://doi.org/10.6084/m9.figshare.29376197).

Additionally, 68 metagenomic sequencing samples corresponding to the 7,318 MAGs were retrieved from the National Center for Biotechnology Information (NCBI) under accession number PRJNA1257558, comprising 26 samples from *Blastocystis-*present individuals and 42 control samples. *Blastocystis* presence in the original dataset was determined by PCR amplification of the SSU rRNA gene using primers RD5 and BhRDr, followed by bidirectional Sanger sequencing and subtype confirmation through BLAST and PubMLST analyses. All Blastocystis-positive samples were identified as the subtype ST31 (18, 19).

### 2.2 Taxonomic annotation

Taxonomic classification of the MAGs was performed using the classify_wf module of GTDB-Tk (v2.3.2) with the GTDB (release 214) (20). Based on the classification results, MAGs assigned to the domain Archaea were selected for further analysis.

### 2.3 Construction of a non-redundant archaeal genome catalog

To construct a non-redundant archaeal genome catalog, we first assessed the quality of archaeal MAGs using the predict module of CheckM2 (v1.0.1) (21). MAGs with completeness >50% and contamination < 5% were retained (4).

We then employed the sketch function in Mash (v2.2) (22) to generate reduced representations of archaeal genomes for rapid comparison, followed by the dist function to estimate pairwise distances between genomes. When two MAGs showed a distance of zero, only the one with higher completeness and lower contamination was retained.

Finally, we used the run module of GUNC (v1.0.5) (23) to evaluate potential chimerism in archaeal MAGs, and only those with a clade separation score (CSS) ≤ 0.45 were kept (24). This resulted in the construction of a non-redundant, non-chimeric archaeal genome catalog from the gut microbiota of Tibetan antelopes, which was subsequently used for downstream analyses (25).

### 2.4 Gene prediction and functional annotation

Protein-coding sequences (CDSs) were predicted and annotated using Prokka (v1.14.6) (26), with the parameter “–kingdom Archaea” to include archaeal-specific non-fragmented proteins from the UniProtKB database, and “—rfam” to enable the identification of non-coding RNAs.

Functional annotation of predicted protein-coding genes was performed by aligning sequences against the Kyoto Encyclopedia of Genes and Genomes (KEGG, Release 20230401) database (27) using DIAMOND (v2.1.8.162) (28) with the parameters: –outfmt 6 –min-score 60 –query-cover 70 –max-target-seqs 5 –masking 1.

The same DIAMOND workflow was used to annotate CAZymes against the Carbohydrate-Active enZYmes (CAZy, Release 20230726) database (29), with parameters: –outfmt 6 –min-score 60 –query-cover 50 –max-target-seqs 5 –masking 1.

### 2.5 Construction of the protein catalog

The protein catalog was constructed based on CDSs predicted by Prokka from 463 archaeal MAGs. All protein sequences were clustered using MMseqs2 (v7e2840992948ee89dcc336522dc98a74fe0adf00) (30) with the following parameters: –cov-mode 1 -c 0.8 –kmer-per-seq 80 –min-seq-id 0.5. To evaluate the clustering performance, protein sequences were clustered at different identity thresholds, and the number of unique taxonomic families represented within each cluster was calculated and visualized. To reduce potential contamination, singleton proteins that could not be assigned to any cluster were removed from downstream analyses.

### 2.6 Raw data preprocessing and sample mapping

A total of 68 raw metagenomic datasets were first quality-filtered using Fastp (v0.23.0) (31) to remove low-quality reads. Host-derived sequences were removed by aligning reads to the host reference genome (GCA_000400835.1_PHO1.0) using Bowtie2 (v2.5.0) (32).

For relative abundance estimation, 20 million high-quality reads were randomly subsampled from each sample and mapped to both the non-redundant archaeal genome catalog and gene catalog using Bowtie2 with default parameters. Read counts were normalized to transcripts per million (TPM). The relative abundance of each functional category was calculated based on the TPM values of genes assigned to that function.

### 2.7 Identification of viral sequences carried by gut archaea

To identify potential viral sequences within the gut archaea of Tibetan antelope, contigs longer than 5,000 bp from 463 non-redundant archaeal genomes were screened (24, 33). First, the ratio of viral to host genes on each contig was assessed using CheckV (v1.0.1) (34). Contigs containing more than 10 host genes or where host genes outnumbered viral genes by more than fivefold were excluded. Proviral fragments were also identified using CheckV.

To enhance the comprehensiveness of viral detection, three complementary methods were employed: (1) viral gene-enriched regions as determined by CheckV; (2) sequences predicted by DeepVirFinder (v1.0.19) (35) with scores >0.90 and *P*-values < 0.01; and (3) viral sequences identified by VIBRANT (v1.2.1) (36) using default parameters. Contigs meeting any of these criteria were retained as putative viral sequences.

To remove potential bacterial contamination, candidate viral sequences were screened for bacterial single-copy orthologs using BUSCO (Benchmarking Universal Single-Copy Orthologs) (37) combined with hmmsearch. The BUSCO ratio (number of BUSCOs divided by total gene count) was calculated, and sequences with a ratio ≥5% were discarded.

### 2.8 Viral community classification and clustering analysis

To characterize viral community structure and assign viral operational taxonomic units (vOTUs) to genus- and family-level taxa, pairwise protein sequence comparisons were performed using BLASTN (v2.13.0) with parameters set to an E-value ≤ 1e-5 and a maximum target sequence number of 99,999. Based on these alignments, the proportion of shared genes and average amino acid identity (AAI) between vOTUs were calculated. Following the approach of Nayfach et al. (38), Markov Cluster Algorithm (MCL) was applied for clustering: at the family level, connections with AAI >20% and shared gene content >10% were retained, with an inflation parameter of 1.2; at the genus level, stricter criteria were applied, requiring AAI > 50% and shared gene content > 20%, with an inflation parameter of 2.

### 2.9 Viral taxonomic annotation

Taxonomic annotation of viral genomes was performed by aligning predicted protein sequences against several reference databases, including the Virus-Host DB (May 2024 release), the crAss-like viral protein database, and other publicly available viral protein datasets (39). Alignments were conducted using DIAMOND with a minimum sequence identity of 30%, query and subject coverage thresholds of at least 50%, and a minimum alignment score of 50. For viral genomes encoding fewer than 30 proteins, assignment to a viral family required at least 20% of the proteins to match proteins from the same family. For genomes encoding 30 or more proteins, at least 10 proteins had to match the same family to allow taxonomic classification (33).

Importantly, none of the viral sequences identified in this study could be assigned to any known viral taxa, suggesting that they may represent novel, previously uncharacterized viral lineages.

### 2.10 Functional annotation of viral sequences

CDSs within the identified viral contigs were predicted using Prodigal (v2.6.3) (40) with the parameter “-p meta,” optimized for metagenomic data. The resulting protein sequences were then aligned to the KEGG and CAZy databases using the same annotation strategy and parameters described above, in order to identify the potential metabolic and functional profiles encoded by the viral genomes.

### 2.11 Comparative analysis of gut archaeal composition among Tibetan antelope, humans, and chickens

In this study, we compiled a total of 60,664 MAGs from the human gut microbiome (sourced from: https://github.com/snayfach/IGGdb) and 26,053 MAGs from the chicken gut microbiome. The chicken-derived MAGs included 12,339 from the National Microbiology Data Center (NMDC, https://nmdc.cn/icrggc), 979 from the ENA database (project IDs: PRJEB55375 and PRJEB55374), and 6,786 from the Figshare repository (https://dx.doi.org/10.6084/m9.figshare.24901884, https://dx.doi.org/10.6084/m9.figshare.24901878, https://doi.org/10.6084/m9.figshare.24681096.v1). Additionally, 5,949 MAGs were obtained from the NCBI database (project ID: PRJNA1099794).

All human- and chicken-derived MAGs were taxonomically annotated using the same classification pipeline applied to the Tibetan antelope MAGs. Quality filtering and dereplication procedures followed the same criteria used to construct the non-redundant archaeal genome catalog of Tibetan antelope. The resulting archaeal MAGs were then used for comparative analysis of gut archaeal species composition across the three host types.

### 2.12 Statistical analysis and visualization

Phylogenetic trees were visualized using the iTOL platform (https://itol.embl.de) (41). Genome structure and target gene annotations were visualized using the CGView platform (https://proksee.ca/) (42). All statistical analyses were conducted in the R environment (v4.2.1). The heatmap of protein distribution was generated using the “pheatmap” package (v1.0.12) (43). To explore differences in protein composition among archaeal taxonomic groups, non-metric multidimensional scaling (NMDS) was performed for visualization. Based on the presence/absence matrix of protein clusters, Bray-Curtis dissimilarity was calculated using the vegdist function from the “vegan” package (v2.6-4) (44), and NMDS ordination was conducted using the metaMDS function. Stress values were reported for each NMDS plot to evaluate the goodness of fit. The statistical significance of compositional differences among groups was assessed using permutational multivariate analysis of variance (PERMANOVA, 999 permutations) via the adonis2 function in vegan. Rarefaction curves were generated using the R package “vegan.” The Observed index, Chao1 index, and Shannon index were calculated based on relative abundance data. beta-diversity was assessed using Bray-Curtis distances and visualized via principal coordinate analysis (PCoA). Differences between groups were tested using PERMANOVA with 999 permutations to assess statistical significance and quantify the proportion of explained variance. Pairwise comparisons between groups were conducted using the Wilcoxon rank-sum test, with a significance threshold set at *P* < 0.05. All other visualizations were performed using “ggplot2” (v3.3.6) (45).

## 3 Results

### 3.1 Non-redundant archaeal genome catalog from the gut microbiota of Tibetan antelopes

**Figure 1:**
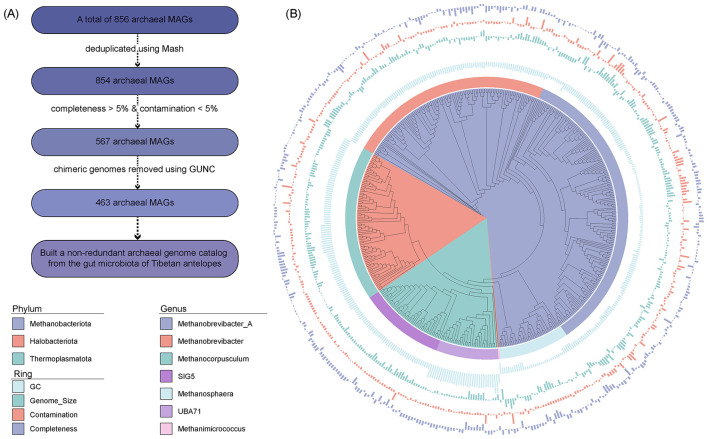
Construction and phylogenetic overview of the non-redundant archaeal genome catalog. **(A)** Workflow for constructing the non-redundant archaeal genome catalog. **(B)** Phylogenetic tree of the non-redundant archaeal genomes. Branches are color-coded by phylum-level taxonomy. The first outer ring represents genus-level classification. The second outer ring shows GC content, with the central line indicating the average across all genomes. The third ring displays genome size, with the central line indicating the average. The fourth ring indicates genome contamination levels, and the fifth ring shows genome completeness, both with central lines representing the corresponding averages across all genomes.

A total of 33,925 MAGs were recovered from the gut microbiome of Tibetan antelopes. Among these, 856 archaeal MAGs were identified based on taxonomic annotation. To remove redundancy, we applied Mash-based clustering and obtained 854 non-redundant archaeal MAGs (Figure 1A). We then filtered for medium-quality MAGs with >50% completeness and < 5% contamination, resulting in 567 MAGs. After further removal of chimeric genomes using GUNC, we obtained a final set of 463 non-redundant, non-chimeric archaeal genomes from the Tibetan antelope gut microbiota.

Taxonomically, these MAGs were primarily assigned to the phylum Methanobacteriota (65.22%), followed by Halobacteriota (17.93%) and Thermoplasmatota (16.85%; Figure 1B; Supplementary Table 1). At the genus level, the dominant groups included *Methanobrevibacter_A* (33.91%), *Methanobrevibacter* (23.33%), *Methanocorpusculum* (17.71%), SIG5 (9.72%), *Methanosphaera* (7.99%), and UBA71 (7.13%). The MAGs exhibited a GC content ranging from 26 to 62% (mean: 39%), and genome sizes from 0.53 Mb to 2.65 Mb (mean: 1.60 Mb). The completeness ranged from 50.55 to 99.97% (mean: 82.30%), while contamination levels varied from 0 to 4.96% (mean: 1.00%).

### 3.2 Protein clustering analysis of gut archaea in Tibetan antelope

**Figure 2:**
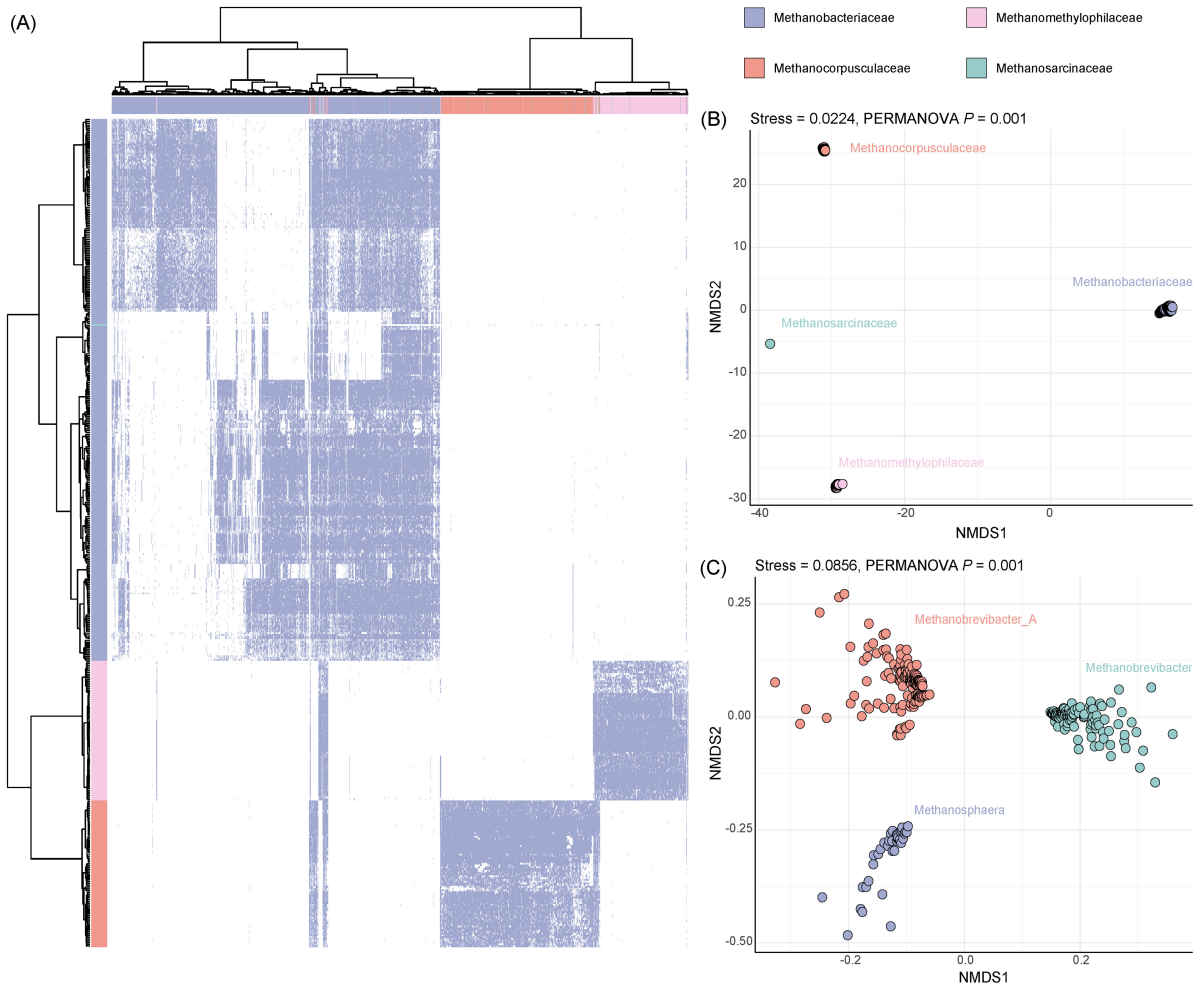
Genomic distribution of archaeal MAGs and the unified protein catalog in the gut of Tibetan antelope. **(A)** A unified protein catalog of archaeal MAGs was constructed based on clustering of all predicted proteins from 463 archaeal genomes using MMseqs2 with thresholds of 50% amino acid identity and 80% alignment coverage. The heatmap displays the presence/absence patterns of 4,027 proteins (columns) across 463 archaeal MAGs (rows); white indicates missing or unavailable data. **(B)** Non-metric multidimensional scaling (NMDS) plot based on the presence/absence matrix of the proteins used in panel A, illustrating separation at the family level. **(C)** NMDS plot showing genus-level differentiation within Methanobacteriaceae. Bray–Curtis distance was used to calculate dissimilarity in **(B)** and **(C)**. Stress values are indicated on each plot. *P*-values were determined by permutational multivariate analysis of variance (PERMANOVA).

A total of 691,494 protein sequences were predicted from 463 archaeal MAGs recovered from the gut microbiota of Tibetan antelopes. These proteins were clustered based on >50% amino acid identity and >80% alignment coverage. Singleton clusters containing only one sequence were removed, resulting in 16,189 representative protein clusters. These clusters collectively formed a non-redundant protein catalog for archaeal genomes. Among them, 4,027 clusters were found to be shared across more than 50 MAGs, suggesting the presence of widely distributed proteins among the archaeal genomes. These shared proteins were primarily derived from *Methanobacteriaceae*, followed by *Methanomethylophilaceae* and *Methanocorpusculaceae*, with only a few from *Methanosarcinaceae* (Figure 2A). To assess differences in protein composition across archaeal phylogenetic groups, we performed non-metric multidimensional scaling (NMDS) analysis. The results showed that the four dominant archaeal families-*Methanobacteriaceae, Methanomethylophilaceae, Methanocorpusculaceae*, and *Methanosarcinaceae* were clearly separated in the NMDS space (Figure 2B), indicating notable differences in their protein composition. Given the predominance of *Methanobacteriaceae* in the archaeal community, we further examined genus-level variation within this family. NMDS analysis revealed a clear separation among the genera *Methanobrevibacter_A, Methanobrevibacter*, and *Methanosphaera* (Figure 2C). Notably, although *Methanobrevibacter_A* and *Methanobrevibacter* differ only by a suffix in name, they are classified as distinct genera according to the GTDB-Tk taxonomy, which is consistent with the observed differences in their protein composition.

### 3.3 Alterations in gut archaeal diversity and composition associated with *Blastocystis* presence

*Blastocystis* is a protist widely present in the intestines of humans and animals, and its presence may lead to alterations in the structure and function of the host gut microbiota. To determine whether *Blastocystis presence* affects the diversity and composition of gut archaea in Tibetan antelopes, we compared the archaeal communities between the *Blastocystis*-present (BP) group and healthy controls (HC) group.

**Figure 3:**
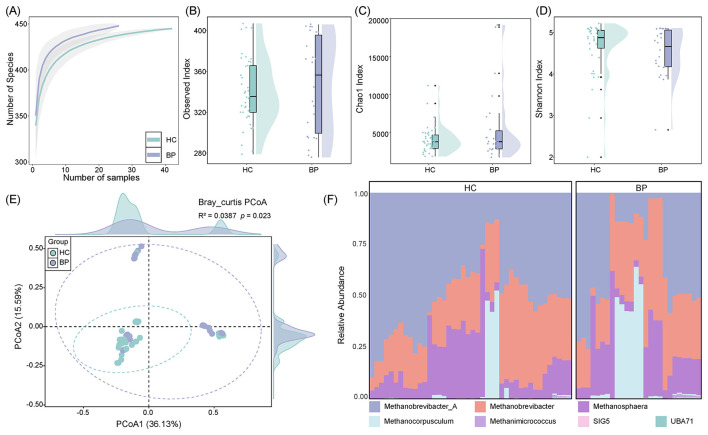
Effects of *Blastocystis* presence on the diversity and composition of gut archaeal communities. **(A)** Rarefaction curves illustrating the relationship between gene relative abundance accumulation and increasing sample size. **(B)** Boxplot of the Observed Index showing differences in the number of observed archaeal species between the HC (healthy control) and BP (*Blastocystis-*present) groups. **(C)** Boxplot of the Chao1 Index estimating community richness between the HC and BP groups. **(D)** Boxplot of the Shannon Index representing differences in community diversity and evenness between the two groups. **(E)** Principal coordinates analysis (PCoA) based on beta-diversity, displaying sample distribution along the first two principal coordinates (PCoA1 and PCoA2), with the percentage of explained variance labeled on each axis. Ellipses indicate 95% confidence intervals for each group. Density plots on the top and right panels represent sample distribution along PCoA1 and PCoA2, respectively. **(F)** Stacked bar plot showing the relative abundance of gut archaeal taxa at the genus level in the HC and BP groups.

Rarefaction curve analysis indicated that the archaeal richness in both groups approached saturation with increasing sequencing depth (Figure 3A), suggesting adequate coverage of archaeal diversity. Comparisons of alpha diversity indices, including the Observed (Figure 3B), Chao1 (Figure 3C), and Shannon (Figure 3D) indices, showed no significant differences between the two groups (*P* > 0.05). However, principal coordinates analysis (PCoA) revealed a significant separation in community structure between the BP and HC groups (Figure 3E, *R*^2^ = 0.0387, *P* = 0.023).

At the phylum level, the archaeal communities in both groups were dominated by Methanobacteriota (HC: 96.13%, BP: 87.56%), followed by Halobacteriota (HC: 3.80%, BP: 12.26%) and Thermoplasmatota (HC: 0.07%, BP: 0.17%; Supplementary Figure 1A; Supplementary Table 2). Notably, the relative abundance of Methanobacteriota was significantly reduced in the BP group (*P* < 0.01), while the proportions of Halobacteriota and Thermoplasmatota were significantly increased (*P* < 0.01; Supplementary Figures 1B, C).

At the genus level, the dominant archaeal taxa in both groups included *Methanobrevibacter_A* (HC: 51.97%, BP: 35.80%), *Methanobrevibacter* (HC: 28.12%, BP: 33.25%), and *Methanosphaera* (HC: 16.04%, BP: 18.51%; Figure 3F). Notably, *Methanobrevibacter_A* was significantly decreased in the BP group (*P* < 0.05), whereas *Methanocorpusculum* (*P* < 0.05), SIG5 (*P* < 0.01), and UBA71 (*P* < 0.01) were significantly enriched following *Blastocystis* presence (Supplementary Figure 2).

### 3.4 *Blastocystis* presence alters the functional profile of gut archaea

To investigate the functional characteristics of gut archaea in Tibetan antelopes and assess the potential impact of *Blastocystis* presence, we performed functional annotation and comparative analysis based on the KEGG and CAZy databases.

**Figure 4:**
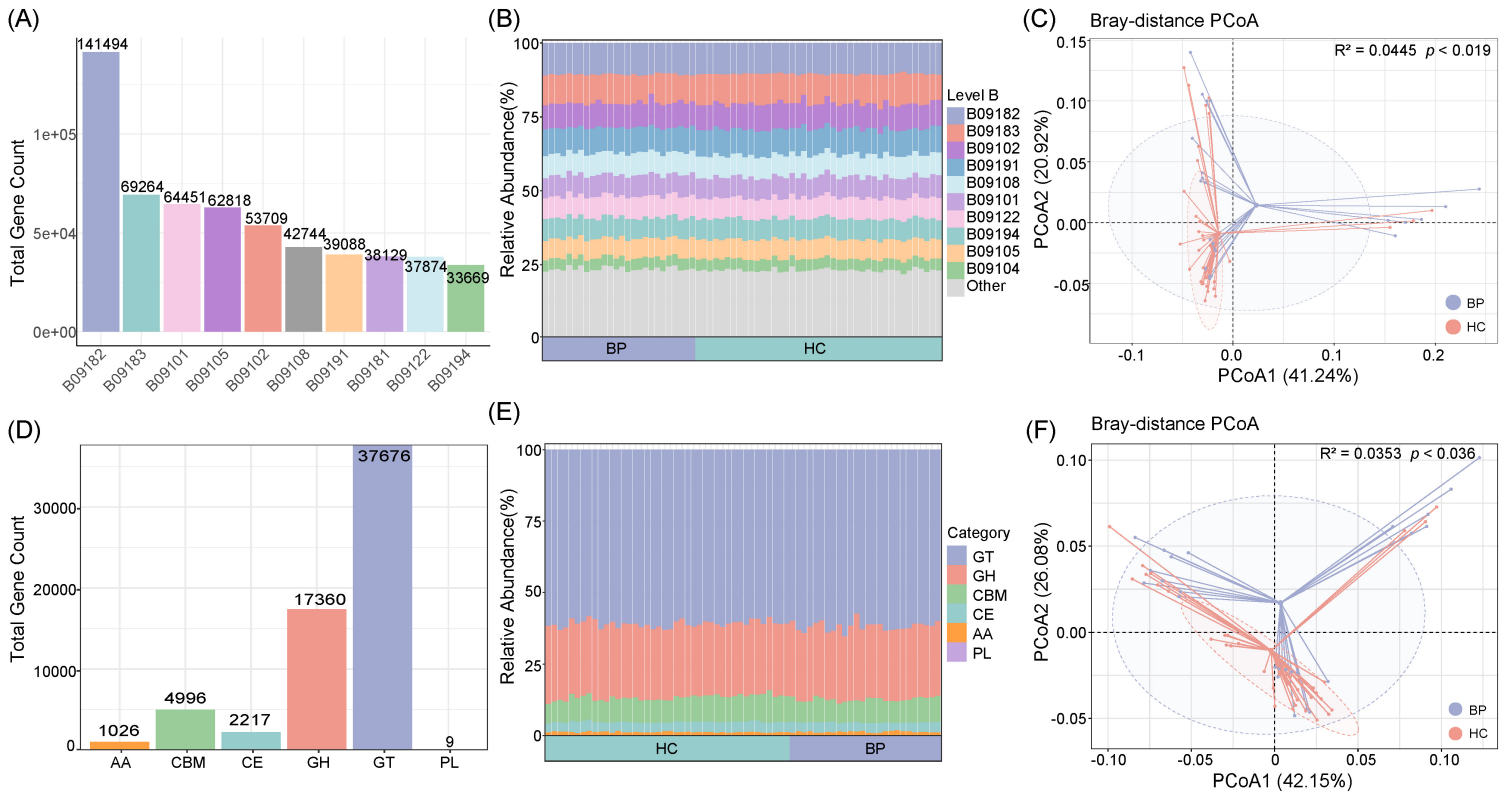
Functional profiling of gut archaeal communities in Tibetan antelopes. **(A)** Bar chart showing the number of genes annotated to KEGG level-B functional categories. Only the top 10 most abundant categories are shown. **(B)** Stacked bar plot displaying the relative abundance of KEGG level-B functional categories. Only the top 10 categories are shown; remaining functions are grouped as “Other.” **(C)** Principal coordinates analysis (PCoA) based on KEGG functional profiles, illustrating differences between the *Blastocystis-*present (BP) and healthy control (HC) groups. Samples are plotted along the first two principal coordinates (PCoA1 and PCoA2), with the percentage of explained variance indicated. Ellipses represent 75% confidence intervals. **(D)** Bar chart showing the number of genes annotated to different CAZy functional modules, including GH (Glycoside Hydrolases), GT (GlycosylTransferases), PL (Polysaccharide Lyases), CE (Carbohydrate Esterases), CBM (Carbohydrate-Binding Modules), and AA (Auxiliary Activities). **(E)** Stacked bar plot illustrating the relative abundance of CAZy functional modules. **(F)** PCoA plot based on CAZy functional profiles, showing differences between the BP and HC groups. Samples are distributed along PCoA1 and PCoA2, with variance explanation percentages indicated on each axis. Ellipses represent 75% confidence intervals.

For KEGG annotation, a total of 445,954 genes were assigned to 2,280 KEGG Orthologs (KOs). Among these, 141,494 genes were associated with Protein families: Genetic Information Processing (B09182), 69,264 with Protein families: Signaling and Cellular Processes (B09183), and 64,451 with Carbohydrate Metabolism (B09101; Figure 4A). In terms of relative abundance, archaeal functions were predominantly related to genetic information processing, signaling, and energy metabolism (Figure 4B). PCoA analysis revealed a significant shift in KEGG-based functional profiles between BP and HC groups (Figure 4C; *R*^2^ = 0.0445, *P* = 0.019). Specifically, Among the functional categories with the highest relative abundances, Energy Metabolism (*P* < 0.01), Nucleotide Metabolism (*P* < 0.05), and Unclassified: Metabolism (*P* < 0.01) were significantly reduced in the BP group, while the relative abundance of Translation was significantly increased (*P* < 0.05; Supplementary Figure 3).

For CAZy annotation, 63,284 genes were assigned to 199 CAZyme families. Of these, 37,676 genes were related to GlycosylTransferases (GT), 17,360 to Glycoside Hydrolases (GH), 4,996 to Carbohydrate-Binding Modules (CBM), 2,217 to Carbohydrate Esterases (CE), 1,026 to Auxiliary Activities (AA), and 9 to Polysaccharide Lyases (PL; Figure 4D). In terms of relative abundance, GTs accounted for more than 50% of the archaeal carbohydrate-active enzymes (CAZymes), followed by GHs and CBMs (Figure 4E). PCoA analysis further indicated a significant alteration in the carbohydrate metabolic potential of archaea between the BP and HC groups (Figure 4F). Specifically, the relative abundances of CBM (*P* < 0.01), CE (*P* < 0.05), and PL (*P* < 0.05) were significantly decreased in the BP group, while the relative abundance of GT was significantly increased (*P* < 0.05; Supplementary Figure 4).

### 3.5 Potential viruses associated with gut archaea

To investigate the presence of archaeal viruses in the gut microbiota of Tibetan antelopes, we performed virus identification based on the constructed non-redundant archaeal genome catalog. As a result, seven viral sequences were identified, all of which were unclassified viruses. Functional annotation revealed that two viral sequences were assigned to K20276 (*bapA*), which encodes a large repetitive protein involved in the quorum sensing pathway (ko02024). In addition, five viral sequences were annotated as members of the glycosyltransferase family 4 (GT4), which are commonly associated with the synthesis and modification of polysaccharides.

### 3.6 Distinct composition of gut archaea in Tibetan antelopes

To explore the compositional characteristics of gut archaea in Tibetan antelopes, we compared the taxonomic distribution of their non-redundant archaeal genomes (MAGs) with those from humans and chickens. At the phylum level, archaeal MAGs from Tibetan antelopes were assigned to three phyla: Methanobacteriota (65.23%), Halobacteriota (17.93%), and Thermoplasmatota (16.85%; Supplementary Figure 5A). In contrast, the majority of archaeal MAGs from humans belonged to Methanobacteriota (81.87%), with much lower proportions of Halobacteriota (1.55%) and Thermoplasmatota (16.58%; Supplementary Figure 5B). In chickens, Halobacteriota was the dominant phylum (52.02%), followed by Thermoplasmatota (22.54%) and Methanobacteriota (25.43%; Supplementary Figure 5C).

**Figure 5:**
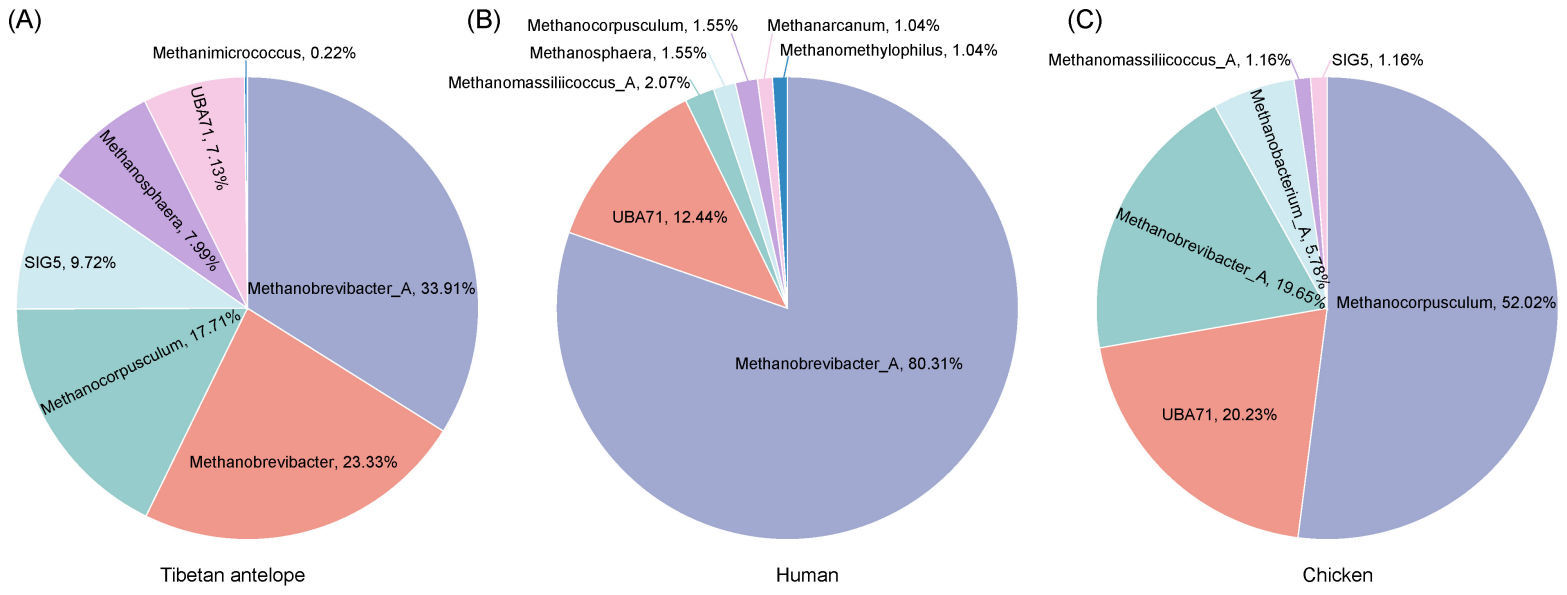
Comparative analysis of archaeal species composition in the gut microbiota of Tibetan antelopes, humans, and chickens. **(A)** Number of archaeal MAGs assigned to each phylum in the gut microbiota of Tibetan antelopes. **(B)** Number of archaeal MAGs assigned to each phylum in the human gut microbiota. **(C)** Number of archaeal MAGs assigned to each phylum in the chicken gut microbiota.

At the genus level, the predominant archaeal genera in Tibetan antelopes were *Methanobrevibacter_A* (33.91%), *Methanobrevibacter* (23.33%), and *Methanocorpusculum* (17.71%; Figure 5A). In humans, *Methanobrevibacter_A* dominated (80.31%), followed by UBA71 (12.44%) and *Methanomassiliicoccus_A* (2.07%; Figure 5B). In chickens, the dominant genera were *Methanocorpusculum* (52.02%), UBA71 (20.23%), and *Methanobrevibacter_A* (19.65%; Figure 5C).

Notably, 71.50% of archaeal MAGs from Tibetan antelopes could not be classified to known species, whereas the proportion of unclassified MAGs at the species level was only 1.55% in humans and 1.73% in chickens (Supplementary Table 4), indicating a higher degree of novelty and unexplored diversity in the gut archaeal community of Tibetan antelopes.

## 4 Discussion

This study employed metagenomic approaches to reconstruct a non-redundant and non-chimeric gene catalog comprising 463 archaeal MAGs from the gut microbiota of Tibetan antelopes. It systematically revealed the diversity, phylogenetic characteristics, and alterations in community structure and functional profiles of gut archaea in response to *Blastocystis* presence, providing key insights into the archaeal ecology of wild animals inhabiting the plateau.

In this archaeal genome catalog, over 70% of the archaeal MAGs from the gut microbiota of Tibetan antelopes could not be classified into any known species, highlighting the high novelty and unexplored diversity of archaea in this high-altitude host. In contrast, archaeal populations in humans and chickens exhibit much lower novelty, with fewer than 2% of MAGs representing potentially novel species. This stark difference suggests that the unique environmental pressures of the plateau may have driven the development of distinct archaeal symbioses in Tibetan antelopes (1, 3). These findings are consistent with the “host–environment–microbiota co-evolution” theory, which proposes that extreme environments can shape specialized microbial niches and accelerate species divergence (3, 7, 46, 47). As a flagship species of the high-altitude ecosystem, the Tibetan antelope and its gut archaeome offer a valuable model for investigating microbial evolution and ecological adaptation in plateau environments.

Although no significant differences were observed in archaeal alpha-diversity indices between *Blastocystis-*present Tibetan antelopes and healthy controls, PCoA analysis revealed a clear separation in archaeal community structure between the two groups. Although *Blastocystis* has been associated with gastrointestinal symptoms in some hosts, its biological role remains controversial, with evidence supporting both commensal and opportunistic pathogenic lifestyles (10). The observed stability in archaeal alpha-diversity, together with the compositional shifts, suggests that *Blastocystis* may engage in specific interactions with archaeal taxa rather than inducing broad gut dysbiosis in Tibetan antelopes. At the phylum level, Methanobacteriota dominated in both groups but showed a significant decrease in relative abundance in the BP group, whereas Halobacteriota and Thermoplasmatota were significantly enriched. This shift may indicate that methanogenic archaea such as Methanobacteriota are suppressed in response to protozoan presence, while other archaeal taxa expand to adapt to the altered gut environment (14, 48). At the genus level, *Methanobrevibacter_A, Methanobrevibacter*, and *Methanosphaera* were dominant in both groups, but *Methanobrevibacter_A* significantly decreased in the BP group, whereas several low relative abundance genera, including *Methanocorpusculum*, SIG5, and UBA71, showed marked increases in relative abundance. These alterations may be associated with *Blastocystis*-induced gut inflammation, shifts in metabolic substrates, or host immune modulation (14, 49), further supporting the possibility that specific archaeal lineages differentially respond to protozoan colonization and indicating that these rare archaea may play potential roles in host responses to protozoan presence, warranting further investigation.

Functional annotation revealed that the gut archaea of Tibetan antelope possess extensive metabolic potential, and *Blastocystis* presence significantly altered archaeal metabolic profiles across multiple functional modules. KEGG-based analysis showed that archaeal functional genes were mainly enriched in pathways related to genetic information processing, signal transduction, and energy metabolism, reflecting their essential roles in maintaining host gut microbial homeostasis (50–52). In the BP group, the relative abundances of energy metabolism, nucleotide metabolism, and “unclassified metabolism” pathways were significantly decreased, suggesting that *Blastocystis* presence may suppress the energy production and basal metabolic capacity of archaea. Because methanogens consume microbial hydrogen, their functional decline may increase luminal hydrogen levels, subtly shifting gut redox conditions in ways that could facilitate *Blastocystis* persistence (10, 53). In contrast, the upregulation of translation-related genes may represent a stress response mechanism to maintain protein synthesis and functional integrity (14, 54, 55). CAZy annotation further revealed that *Blastocystis* presence disrupted the carbohydrate metabolic potential of archaea. GTs accounted for over 50% of archaeal CAZymes, indicating a strong capacity for polysaccharide synthesis and structural modification (51, 56). Following presence, the relative abundance of GTs significantly increased, while that of CBMs, CEs, and PLs significantly decreased. This imbalance may impair archaea's ability to acquire carbon sources and degrade polysaccharides. The reduction in CBMs suggests a decreased capacity to bind complex carbohydrates such as cellulose, while decreased CE and PL relative abundances may lower the efficiency of polysaccharide degradation, potentially affecting host energy acquisition and gut nutrient metabolism (56, 57). These reductions in archaeal carbohydrate-degrading functions may alter nutrient fluxes in the gut microbiome, potentially creating ecological conditions more favorable to *Blastocystis*. In addition, although the identified viral sequences could not be classified into known viral taxa, their predicted functions involved quorum sensing and polysaccharide metabolism, suggesting that these viruses may participate in mediating archaeal interactions or influencing host-microbiota relationships (58–60).

This study revealed significant impacts of *Blastocystis* presence on the structure and function of the gut archaeal community in Tibetan antelopes. However, the findings are limited by sample type and the incomplete coverage of current reference databases. Future studies should incorporate longitudinal sampling and experimental validation to further elucidate the specific roles of archaea in host responses to *Blastocystis* presence.

## 5 Conclusion

In this study, we employed a metagenomic approach to systematically investigate the composition, diversity, and functional alterations of the gut archaeal community in Tibetan antelopes in the presence of *Blastocystis*. We constructed a genome catalog comprising 463 non-redundant archaeal MAGs, covering 16,189 protein clusters, over 70% of which could not be classified to any known species, highlighting the high novelty and unexplored diversity of gut archaea in this high-altitude host. Community analysis revealed that although *Blastocystis* presence had no significant effect on archaeal alpha diversity, it caused marked shifts in community structure, characterized by a significant enrichment of Halobacteriota and Thermoplasmatota, and a reduction in Methanobacteriota. Functional annotation further showed that presence significantly perturbed archaeal pathways involved in energy metabolism, nucleotide metabolism, and carbohydrate-active enzyme composition, suggesting that *Blastocystis* presence may influence host health by reshaping the metabolic network of the gut microbiota. Although only a small number of viral sequences were detected, all of which were unclassified archaeal viruses, underscoring the largely unexplored viral diversity associated with the archaeal community in this high-altitude host. Collectively, this study fills a critical gap in our understanding of the archaeal ecology of wild animals in high-altitude environments and expands our knowledge of archaeal diversity, ecological adaptation, and interactions with protists. These findings provide a valuable resource and new perspective for future studies on the microbiome ecology and evolution of plateau wildlife.

## Data Availability

Publicly available datasets were analyzed in this study. This data can be found here: CNGBdb database (Project ID: CNP0001390), Figshare repository, http://doi.org/10.6084/m9.figshare.29376197, National Center for Biotechnology Information (NCBI) under accession number PRJNA1257558.
