# Supplementary material for: Blastocystis presence alters gut archaeal communities and metabolic functions in Tibetan antelopes (Pantholops hodgsonii)
Source: Front Vet Sci. 2025 Dec 23;12:1744013. doi: 10.3389/fvets.2025.1744013 (PMC12771539; doi:10.3389/fvets.2025.1744013)
Supplement: Supplementary file 2 [file Data_Sheet_1.docx]

**Supplementary Figures:**


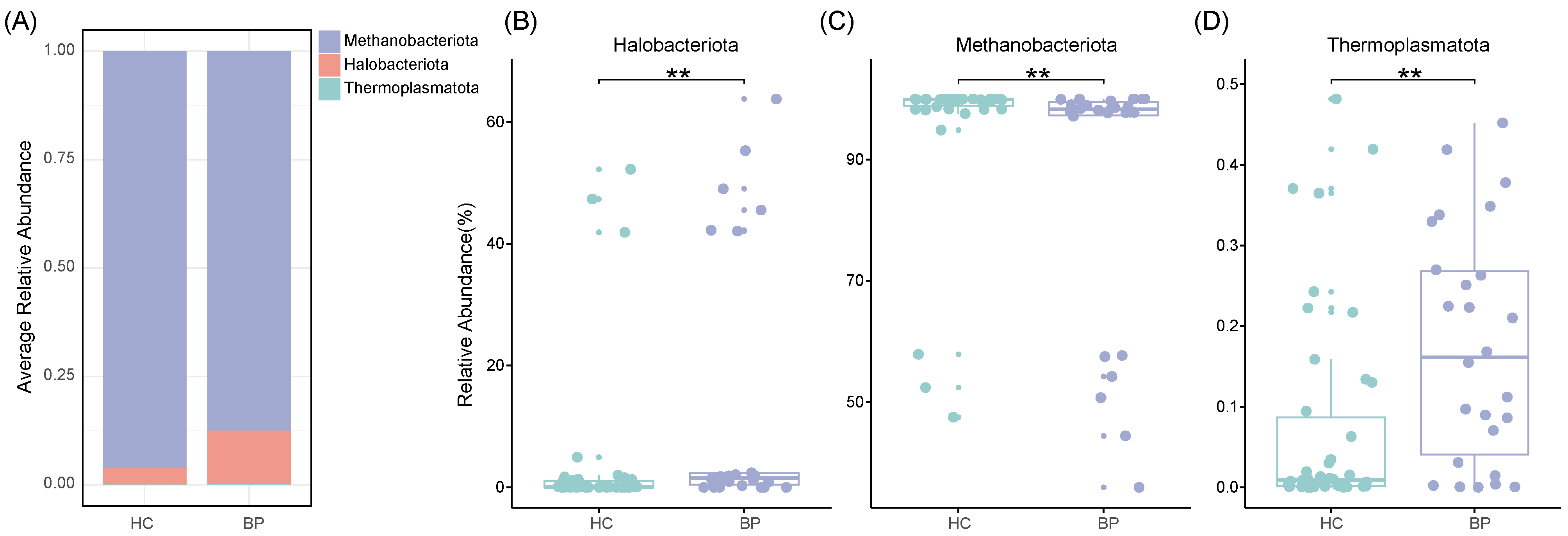


**Supplementary Fig. 1: Phylum-level compositional features of gut archaeal communities.** (A) Relative abundance of archaeal phyla in the gut microbiota of healthy controls (HC) and *Blastocystis-*present (BP) groups. (B-C) Box plots showing the differences in relative abundance of archaeal phyla between the two groups. Statistical significance was assessed using the Wilcoxon rank-sum test: **P* < 0.05; ***P* < 0.01.


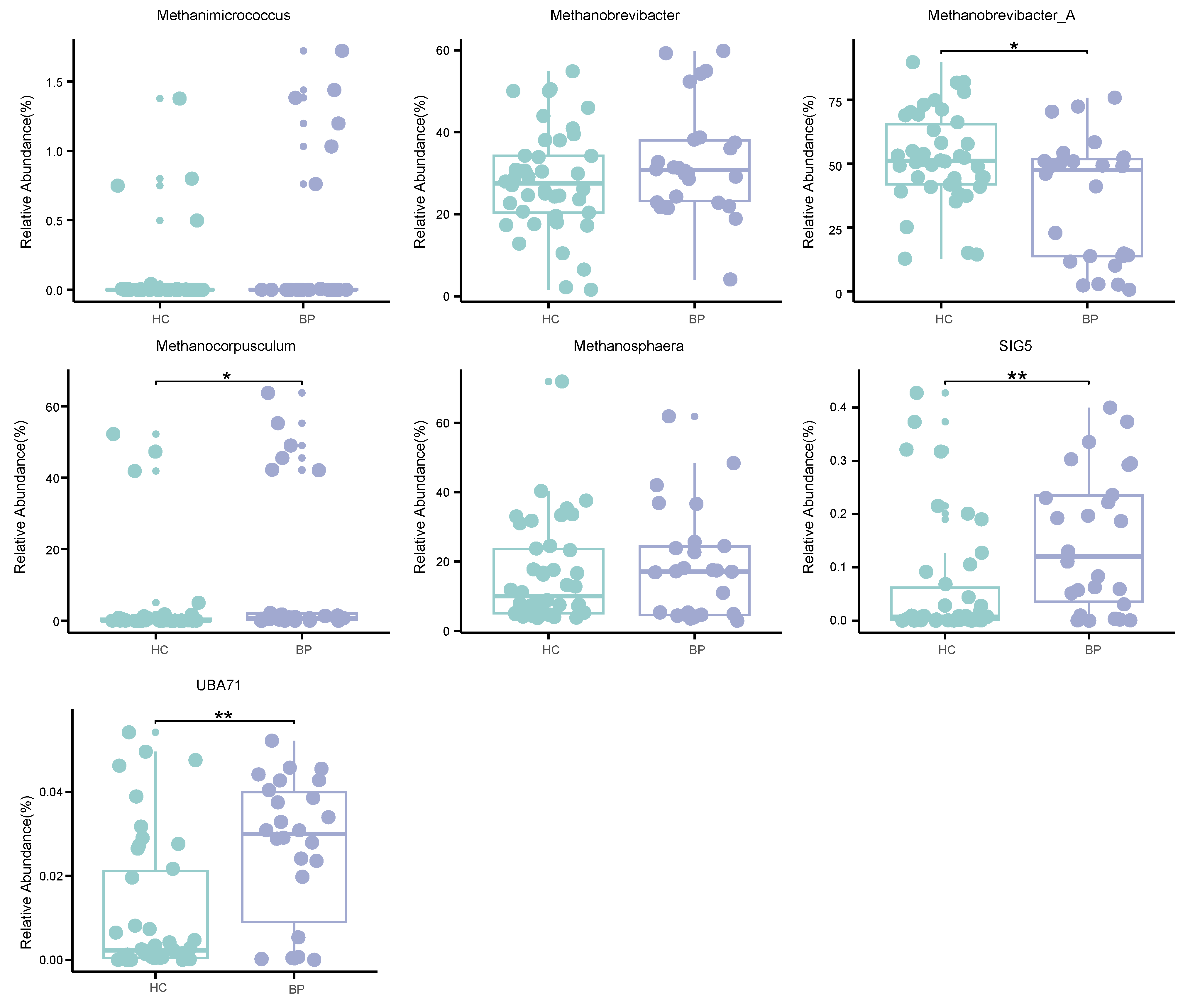


**Supplementary Fig. 2: Differential abundance of gut archaeal genera between *Blastocystis-*present （BP）and healthy control (HC) groups.** Box plots show differences in the relative abundance of archaeal genera between the two groups. Statistical significance was assessed using the Wilcoxon rank-sum test: **P* < 0.05; ***P* < 0.01.

**
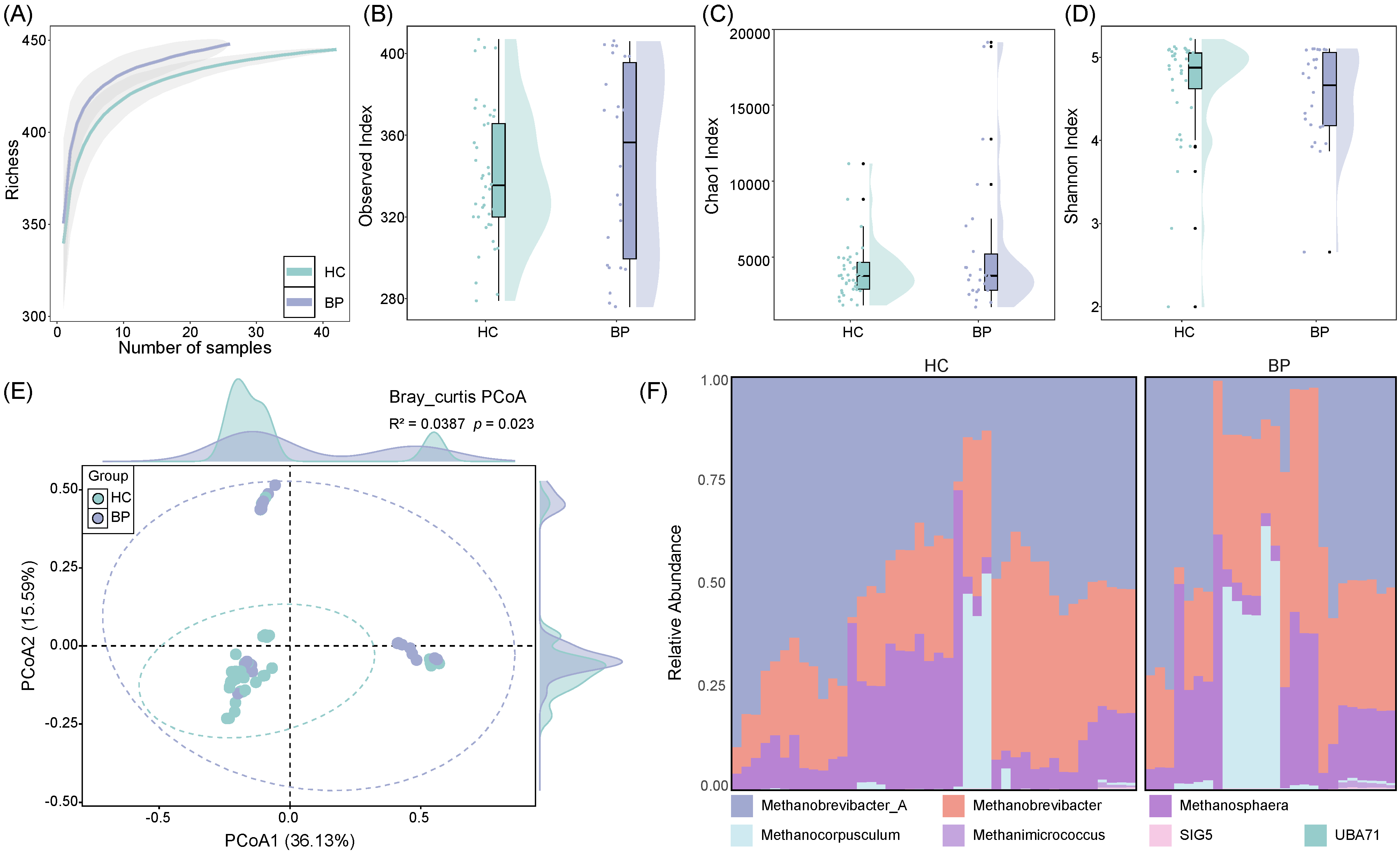
**

**Supplementary Fig. 3: Functional differences between *Blastocystis-*present (BP) and healthy control (HC) groups.** Box plots show the relative abundance differences of the top ten KEGG level-B functions between the BP and HC groups. Statistical significance was assessed using the Wilcoxon rank-sum test: **P* < 0.05; ***P* < 0.01.


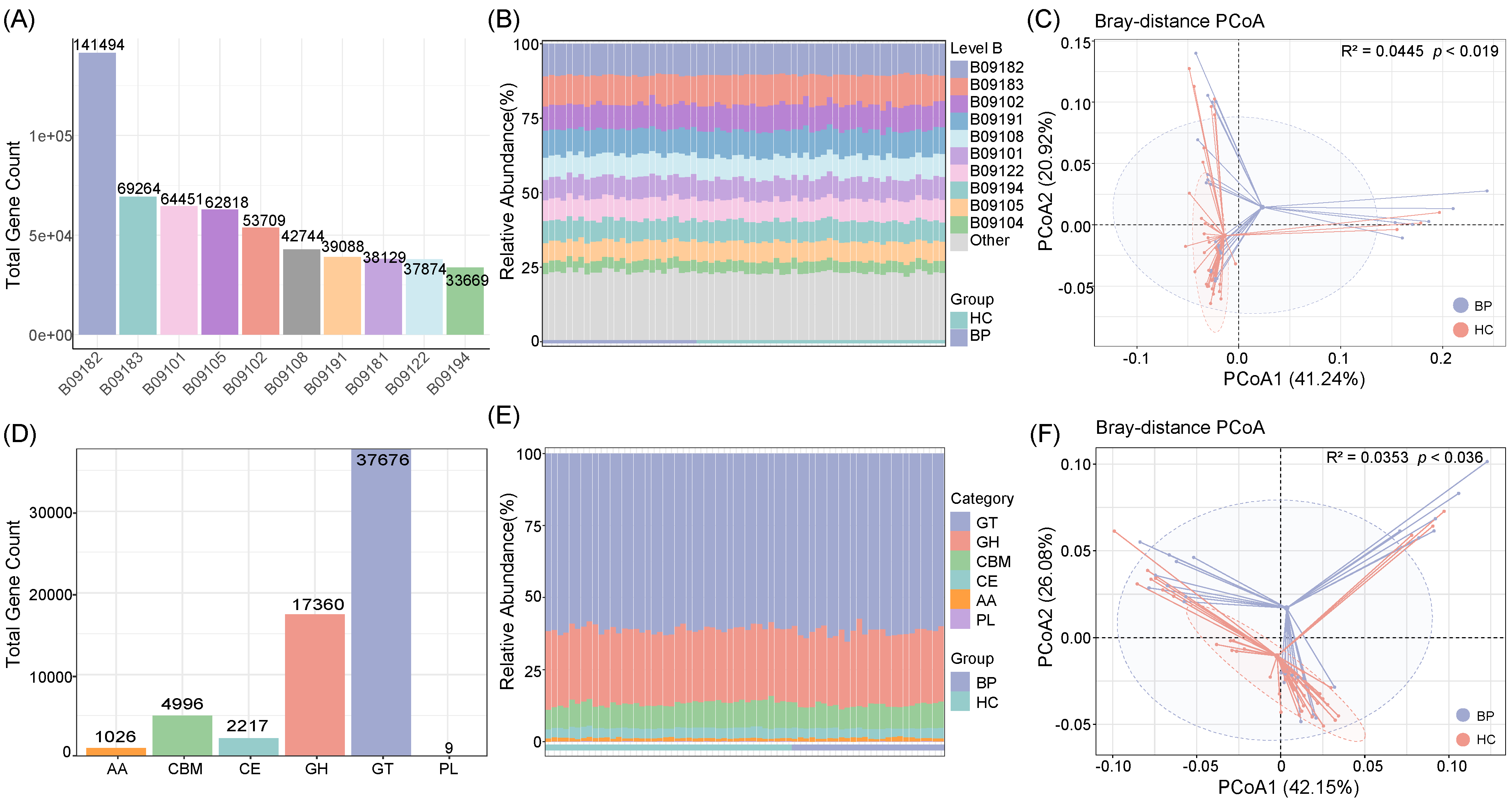


**Supplementary Fig. 4: Differences in carbohydrate-related functions between *Blastocystis*-present (BP) and healthy control (HC) groups.** Box plots illustrate the relative abundance differences of CAZy functional modules between the BP and HC groups, including GH (Glycoside Hydrolases), GT (GlycosylTransferases), PL (Polysaccharide Lyases), CE (Carbohydrate Esterases), CBM (Carbohydrate-Binding Modules), and AA (Auxiliary Activities). Statistical significance was assessed using the Wilcoxon rank-sum test: **P* < 0.05; ***P* < 0.01.


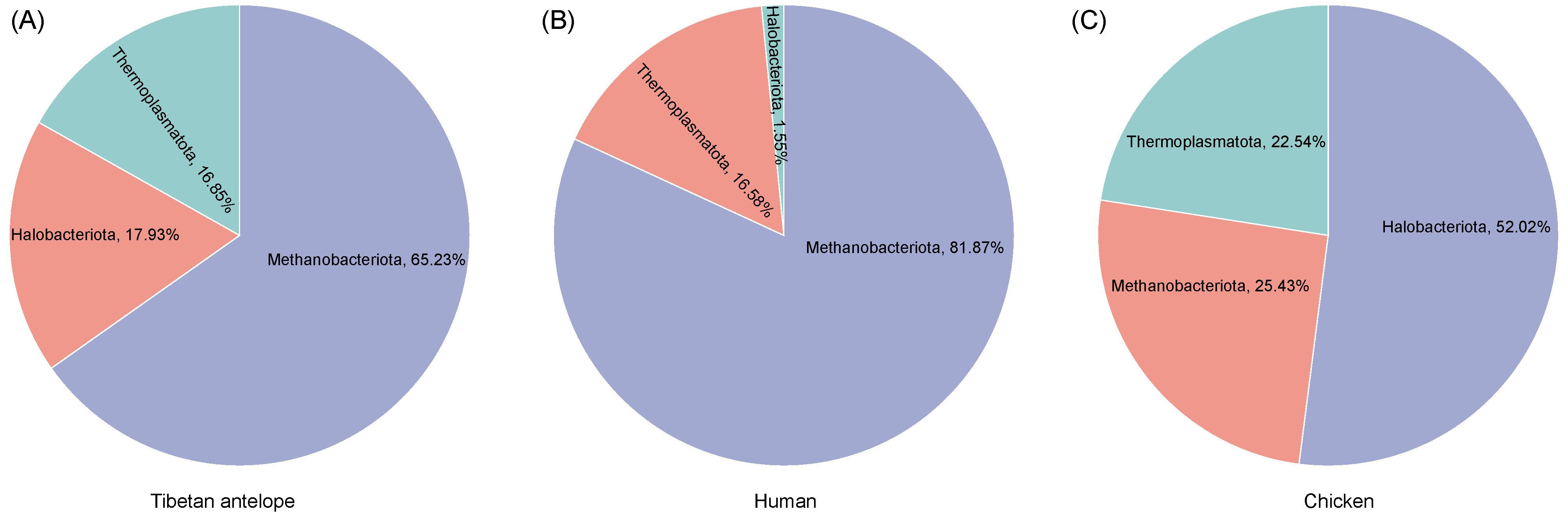


**Supplementary Fig. 5: Comparative analysis of archaeal species composition at the phylum level in the gut microbiota of Tibetan antelopes, humans, and chickens.** (A) Number of archaeal MAGs classified at the phylum level in the gut microbiota of Tibetan antelopes. (B) Number of archaeal MAGs classified at the phylum level in the human gut microbiota. (C) Number of archaeal MAGs classified at the phylum level in the chicken gut microbiota.
